# Management of Cold Water-induced Hypothermia: A Simulation Scenario for Layperson Training Delivered via a Mobile Tele-simulation Unit

**DOI:** 10.7759/cureus.1990

**Published:** 2017-12-26

**Authors:** Cody L Dunne, Michael Parsons

**Affiliations:** 1 Faculty of Medicine, Memorial University of Newfoundland; 2 Emergency Medicine, Memorial University of Newfoundland

**Keywords:** cold water induced hypothermia, simulation-based education, mobile tele-simulation unit, rural healthcare

## Abstract

Newfoundland and Labrador (NL) has one of the highest provincial drowning rates in Canada, largely due to the many rural communities located near bodies of water. Factor in the province’s cold climate (average NL’s freshwater temperature is below 5.4°C)and the prevalence of winter recreational activities among the population, there exists an inherent risk of ice-related injuries and subsequent hypothermia. Oftentimes, these injuries occur in remote/rural settings where immediate support from Emergency Medical Services (EMS) may not be available. During this critical period, it frequently falls on individuals without formal healthcare training to provide lifesaving measures until help arrives.

Training individuals in rural communities plays an important role in ensuring public safety. In recent years, simulation-based education has become an essential tool in medical, marine and first aid training. It provides learners with a safe environment to hone their skills and has been shown to be superior to traditional clinical teaching methods. The following case aims to train laypeople from rural settings in the immediate management of an individual who becomes hypothermic following immersion into cold water.

However, reaching these individuals to provide training can be a challenge in a province with such a vast geography. To assist with overcoming this, the development of a simulation center that is portable between communities (or Mobile Tele-Simulation Unit) has occurred. By utilizing modern technology, this paper also proposes an innovative method of connecting with learners in more difficult to reach regions.

## Introduction

Across Canada, drowning incidents largely occur in non-supervised areas such as lakes, ponds and oceans. Newfoundland and Labrador (NL), the most eastern province of Canada, is populated with many rural communities founded near bodies of water. The proximity of many communities to large bodies of water may contribute to NL having one of the highest per capita drowning rates in the country [1]. Having access to numerous outdoor water environments and a relatively cold climate, the population has a high participation rate in winter recreational activities. When these activities occur near cold water or ice-covered areas, those participating have an increased risk of hypothermia and ice-related injuries [2].

Hypothermia can be defined as having a core body temperature lower than 35°C. However, where core temperature cannot be measured, a combination of the associated symptoms/signs and a recent history of cold exposure (such as water immersion) can be suggestive of the diagnosis [3].

Several methods have been used to categorize the severity of hypothermia by core temperature, associated signs and symptoms, and likely outcome. A common classification system for layperson training is to divide patients into three categories: mild, moderate and severe. Table 1 defines each category and provides several recognizable signs/symptoms for each [4].

**Table 1:** An example of hypothermia classification and associated symptoms/signs related to certain core body temperatures (°C).

Category of hypothermia	Corresponding temperature (°C)	Associated signs and symptoms
Mild	32.2-35.0	Tachycardia, increased blood pressure (BP), dysarthria, tachypnea, shivering thermogenesis, ataxia
Moderate	28.0-32.1	Depression of level of consciousness, hallucinations, cardiac arrhythmias, hypoventilation, hyporeflexia, diminishing shivering
Severe	<28.0	Very low level of consciousness to coma, significant cardiac arrhythmias to asystole, pulmonary edema, no shivering present

The need for a quick response is emphasized if the environmental exposure inducing hypothermia is water immersion or falling through ice. Immersion into cold water accelerates the speed of body temperature decline and introduces additional risk factors for the injured person, including experiencing a cold shock upon initial immersion [5]. This intense, sudden exposure triggers a number of physiological responses which can escalate how quickly one may become unconscious or non-breathing [5]. Therefore, quick recognition and management of a hypothermic patient is critical to their outcome.

As a province with a vast landscape, accessibility and timeliness of Emergency Medical Services (EMS) response varies greatly depending on the location from which the call for help is made. The role of immediate layperson intervention is critical to improve survival outcomes.

The main goal of many medical and first aid training programs is to provide learners with an environment that fosters the development of their clinical skills and judgment. One method used to achieve this goal is simulation-based education (SBE). SBE creates a safe environment where learners are capable of performing procedures and making clinical decisions without the potential adverse outcomes of involving actual patients. It also has been shown to be superior to other clinical instruction methods [6]. This paper presents a case scenario that can be used to run simulations for the public on the short-term recognition and management of cold water-induced hypothermia. The goal of the case is outlined by the learning objectives and should be supplemented with post-simulation didactic teaching reinforcing the principles of self-protection, ice safety and basic aquatic patient recovery.

NL's rural, dispersed population also creates financial and logistical obstacles to providing effective public education programs such as this. This paper proposes a solution to these challenges by discussing an alternative method of training to traditional face-to-face instruction.

Tele-simulation is a relatively recent innovation that has enabled mentors to connect with learners over a distance and provide educational opportunities. Tele-simulation is, as defined by McCoy, et al., “a process by which telecommunication and simulation resources are utilized to provide education, training and/or assessment to learners at an off-site location” [7]. To overcome one of the greatest barriers to providing wide-reaching training, tele-simulation centers need to be transportable between communities. Recent work in the field has been conducted to develop a prototype of a Mobile Tele-Simulation Unit (MTU).

Equipped with telecommunication and simulation resources, the MTU allows portability of the simulation center to many locations increasing the geographical capacity of the proposed educational programs. Although originally designed for deployment to rural communities for medical procedures training, partnering with this project has the potential to overcome the barriers associated with providing layperson training.

## Technical report

Case

A 25-year-old female is found with her boyfriend on the edge of a pond after falling through thin ice and becoming completely submerged. He pulled her from the water but now she is collapsed on the shore. She is soaking wet and wearing several layers of clothing. She is responding to voice but her responses are incoherent. Her boyfriend states that they have been on the shore for over 20 minutes and he has not been able to get her to move. He has no first aid equipment or first aid training and is clearly distressed about his girlfriend’s condition. Our learners are equipped with basic first aid supplies and a cell phone.

To clearly describe the scenario, the Context-Input-Process-Product model is utilized below [8].

Context

The scenario was designed to simulate a rural setting away from major healthcare centers. The aim was to provide learners, who are non-health care rural community members, with the skills required to manage a deteriorating hypothermic patient with minimal first aid training and equipment. The scenario could be modified to alter the difficulty level depending on the learners’ prior experience/knowledge. Some possible modifiable factors include the bystander’s level of cooperation/knowledge, the level of consciousness initially of the injured person or the availability (or lack thereof) of certain first aid supplies.

In-situ training (occurring by an actual pond or in the wilderness) would be ideal as it lets learners experience real factors such as the actual temperature, weather conditions, and minimal backup. However, this simulation was designed to run within a Mobile Tele-Simulation Unit (described in detail in the Introduction and Discussion). The unit connected the learners to a mentor stationed at another location. The simulated persons/patients (SPs) were placed in the center of the unit so that the various video cameras were focused on them and allowed the mentor to adequately see the actions performed by the learners. Proper placement of SPs and learners ensured minimal interruption as the simulation was running – as going off camera/out of microphone distance is one of the limitations of the MTU. During the pre-briefing session, one aim was to prep the learners for functioning within the MTU and its limits. This was done by showing learners the setup and where they could/could not be seen and heard to help minimize disruptions to the facilitator’s assessment of them. This scenario was employed in moderate temperatures to minimize the discomfort of the SP in wet clothing. However, with safety precautions put in place for the learners and SPs, conducting the same scenario in more frigid, realistic temperatures would be possible to prepare learners for what they may have to face.

Inputs

*Personnel*

This simulation included three confederates, all trained simulated persons. According to the Healthcare Simulation Dictionary, an SP is a person who portrays a patient (simulated patient), family member, or healthcare provider in order to meet the objectives of the simulation [9]. In addition, SPs can also engage in assessment by providing feedback to the learner. This is a feature that was not utilized in this scenario but it can be added [10].

The first SP was the bystander boyfriend who, depending on the targeted difficulty level of the scenario, could range from being a helpful assistant to suffering from crippling distress.

The second SP was the hypothermic patient. For added realism, the patient used body paint/coloring (moulage) to simulate poor perfusion and wore wet clothing. This patient SP was instructed when to simulate their level of consciousness change to minimize interruption by the facilitator.

The third SP was the EMS operator who joined the conversation over the phone, and who would theoretically dispatch the ambulance when the learners requested it.

Equipment

The case dictated that basic wilderness first aid equipment is available for the rescuer including: towels/blankets, gloves, and a pocket mask. To increase difficulty, the pocket mask could have been removed to explore the learners’ knowledge of compression-only cardiopulmonary resuscitation (CPR). A CPR mannequin was on hand to facilitate completion of proper Basic Life Support (BLS) protocol. As well, a cell phone was available which the learners’ used to contact EMS.

Process

*Pre-Briefing*

A pre-briefing occurred before each attempt at the simulation. The goal of this session was to prepare the learners for the scenario and explain the limits of the simulation. It is good practice to establish a safe learning environment and discuss a concept coined by Rudolph, et al. as “the basic assumption” [11]. This concept proposes that everyone is intelligent, will try their best and wants to improve from the activity. At this time, the learners are told whether there is an assessment component attached to the simulation. As the aim of this project was for public education, our learners were instructed that this was for skill development only.

The learners were also allowed to explore the MTU prior to the start and shown where the boundaries were for the cameras and microphones. This ensured the facilitator did not miss any actions conducted by the learners during the actual scenario due to inability to see/hear them. The specific SPs/equipment relevant to this simulation were not present in the MTU during the tour in an effort to not reveal the contents/objectives of the training session.

The learners were then provided with the pre-scenario below and allowed to enter the simulation.

*Pre-Scenario*

This stem was used to introduce the learner to the scenario:

You are a visitor to Little Falls, a rural community situated in central NL. Taking advantage of the sunny, but brisk, winter day you decide to take a hike about 15 minutes outside of the town. As the hike takes you past one of the many neighboring ponds you admire the glistening of the fresh snow and ice. While taking in the view, you suddenly notice two individuals by the water’s edge. One of them is clearly in distress and the second does not seem to be moving. Upon approaching them, the young male tells you his girlfriend just fell through the pond’s ice and he had to drag her back to shore. He says that she doesn’t seem herself now and he hasn’t been able to get her moving much for the past 20 minutes. As an experienced hiker, you know some basic first aid and have a few first aid supplies in your bag. You offer to help the couple.

*Scenario*

The scenario started when the learners approached the couple. The boyfriend provided a basic history of what had occurred (the depth of details depended on the SPs instructed level of assistance). He also provided a history as requested by the learners with details on allergies, medications, past medical history, last meal and events associated with the incident (AMPLE history).

**Figure 1:**
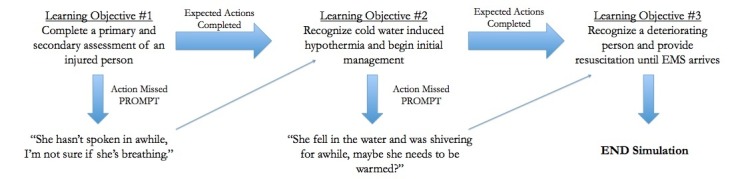
Learners' progression through the simulation scenario: management of a deteriorating hypothermic individual.

Upon initial presentation, the patient was moulaged in a way that the learners should be able to tell (or otherwise be informed in the pre-scenario) that the patient was wet. The patient was responsive to verbal stimulus but incoherent. The learner assessed the patient, performing a primary and secondary assessment. Based on the findings, as outlined in Figure 1, the learners recognized that the patient was most likely suffering from severe hypothermia.

The decreased level of consciousness (LOC) was a trigger for the learners to activate EMS using the cell phone available. They called themselves (alternatively, they could have directed the boyfriend to do so). Appropriate information was provided to the EMS operator. If information was missing, the EMS dispatch SP should have prompted the learners that more information was required.

Once the learners recognized that the patient was severely hypothermic, they initiated rewarming. Multiple acceptable methods existed to achieve this learning objective and based on the resources available included: removing any wet clothing (while respecting the SP’s privacy), providing body heat using themselves or the bystander, and wrapping the patient in warm blankets/towels [12].

After several minutes of attempted rewarming, the patient became unresponsive. This moment was predetermined before the simulation began by telling the SP to do so after a determined time period (alternatively, it could have occurred after a certain signal was made). Once noticed, the learners reassessed the patient’s airway-breathing-circulation (ABC) – using a head-tilt/chin-lift and visual breathing check – and recognized the need for CPR. The SP simulated their non-breathing state by holding their breath while the primary assessment was being conducted. At this time, the CPR mannequin was switched with the patient SP. CPR was continued for five minutes at which time EMS arrived to take over the successful rescue and the scenario ended. The learners should use the basic first aid equipment provided to perform safe, effective CPR (pocket mask and gloves).

If the learners had failed to recognize the patient's conditions and provide adequate management after two action prompts by the SPs, the scenario could have also been stopped at this time. Figure 1 outlines the flow of the simulation case presented in a diagram format.

*Objectives*

Table 2 outlines the list of expected actions associated with each learning outcome, and the findings that the learners should be presented with once completing them. It can be used as a checklist for the mentor to objectively assess the learners' performances.

**Table 2:** Summary of simulation learning objectives and associated expected actions. BLS: Basic life support; CPR: Cardiopulmonary resuscitation; EMS: Emergency medical services; LOC: Level of consciousness; SP: Simulated persons/patients.

Learning objective #1: Complete a primary and secondary assessment of an individual with decreased level of consciousness.		
Expected action	Findings/outcome	Completed (Y/N)
Assess LOC	Patient responds to verbal stimulus but is incoherent.	
Assess airway/breathing	Patient is taking shallow, slow breaths (respiratory rate = 6, hypoventilation).	
Assess circulation	Patient shows signs of poor perfusion (pallor, heart rate = 36). No obvious blood loss.	
AMPLE history	Environmental allergies. Events preceding as described in case description.	
Head to toe examination (Secondary assessment)	Patient shows no signs of trauma but extremities are tinged blue and the victim is soaking wet. The victim is not shivering.	
Learning objective #2: Recognize cold water-induced severe hypothermia and begin initial management.		
Expected action	Findings/outcome	Completed (Y/N)
Call for help/activate EMS	EMS will arrive in 20 minutes.	
Initiate rewarming attempts	No improvement or change of status from victim despite efforts.	
Position victim comfortably	No improvement or change of status from victim despite efforts.	
Bystander SP should prompt learners if they fail to recognize the diagnosis of hypothermia, or if after 2 minutes they fail to initiate management – prompt should be general. For example, “Maybe she’s too cold.” Bystander should prompt a second time after 4 minutes – prompt should be more direct. For example, “I think we need an ambulance.”		
Learning objective #3: Recognize a deteriorating individual and provide appropriate resuscitation efforts until EMS arrives.		
Expected action	Findings/outcome	Completed (Y/N)
Reassessment of patient	Patient is unresponsive. No visual or vocal signs of breathing.	
EMS update	EMS provides an updated Time of Arrival of 5 minutes.	
Obtain/use proper barrier devices		
Initiate BLS protocol	After 5 minutes of effective CPR END SCENARIO.	
End scenario if: After two prompts from the bystander, if the learners do not manage the hypothermia or recognize the need for CPR, EMS should "arrive" and take over the unsuccessful rescue attempt.		

*Debriefing*

The debriefing session included any learners involved as well as any facilitators. It was conducted immediately following the case in a relaxed environment to facilitate open discussion between the parties involved. The equipment from the MTU used in the scenario was removed and replaced with a fold out table and chairs. Audio-video testing was conducted again before the debriefing began. This was to ensure no connectivity issues during the debriefing, which sometimes can become emotionally charged depending on the learners’ performance.

The focus of the session was to review the simulation, discuss areas where the learners faced obstacles or differed from the expected actions and present learning opportunities to learners [13, 14]. Many methods existed to guide the facilitator through the debriefing process. One model that was employed was the Plus/Delta approach [15]. This model allowed learners to first explore how they felt and what they were thinking at critical moments in the simulation and gave them an opportunity to discuss what thought process led them to certain decisions. Topics of interest in this scenario that the facilitators explored included the need to quickly call for help/advance care in a remote setting and the process of managing a deteriorating patient.

*Post-Scenario Didactics*

Predetermined educational topics were included towards the end of the session to solidify learners’ knowledge of both key simulation concepts and related material. As this case was designed to train learners in the recognition and management of cold water-induced hypothermia relevant topics to explore here included ice safety and basic water recovery. Although this was not a course in aquatic rescue or wilderness survival, an overview of key concepts prepared learners for more complex situations that they may encounter. This scenario was designed so that learners came across the patient once they were on shore, but this could have just as easily been set up so that they first spot the patient falling through ice.

Product

The expected outcomes are highlighted by the three simulation learning objectives:

1. Complete a primary and secondary assessment of an individual with decreased level of consciousness.

2. Recognize submersion-induced severe hypothermia and begin initial management.

3. Recognize a deteriorating individual and provide appropriate resuscitation efforts until EMS arrives.

Additionally, a fourth objective should be included to ensure trainees have obtained a well-rounded education and this should be discussed in the post-scenario didactics:

4. Appreciate the various hazards of activities near ice and demonstrate a basic understanding of ice safety/water rescue principles.

## Discussion

This scenario aims to enhance bystander recognition and care in a critical situation that is relevant to many individuals of NL. It uses a simple simulation case to prepare individuals in case they encounter a life-threatening injury that occurs not uncommonly across the province. As noted in the introduction, training opportunities are limited in NL due to the challenges of providing them to such a rural, dispersed population. Innovative ways to still offer public education opportunities such as this case need to be designed in order to maximize the reach of safety training.

**Figure 2:**
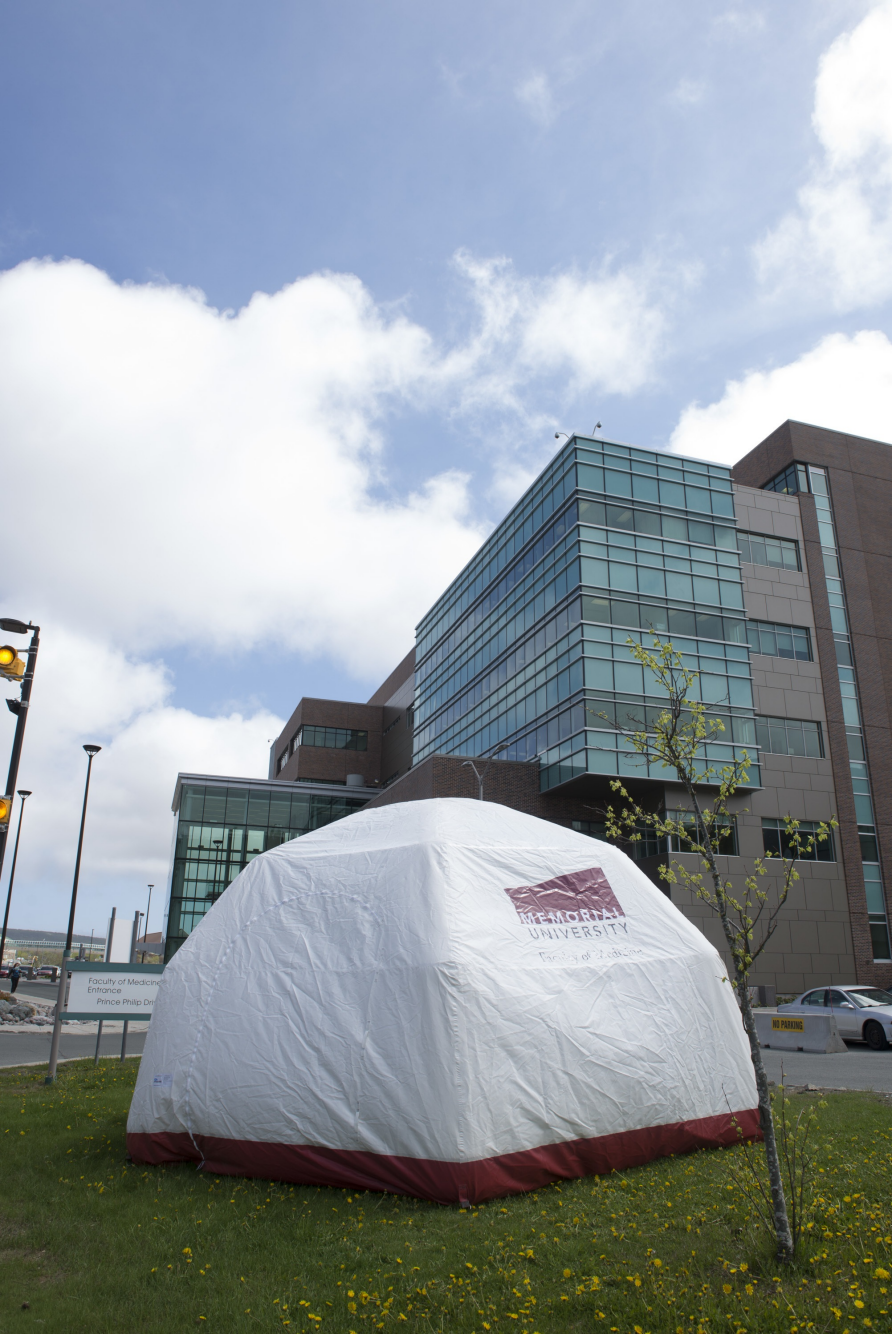
Mobile tele-simulation unit prototype outside Memorial University of Newfoundland (Photo by: HSIMS, MUN).

One innovation under development is an MTU. This portable device is a simulation lab that can be transported anywhere in the province and used to connect learners to facilitators in larger centers through telecommunications (video/audio conferencing) software. The MTU goal is to provide sophisticated training in rural areas without the logistical challenges normal to this type of opportunity (i.e., travel, lack of equipment, cost, etc.). The mobility of the unit further decreases financial constraints on institutions because one unit can be transported to all required training sites as opposed to needing several simulation centers built in rural areas – when they may not be utilized frequently. Figure 2 displays the initial prototype of the MTU's development.

Although originally developed for medical trainees, we propose that the MTU can expand outside the field of medicine and be used as a public education tool. By partnering with communities whose medical professionals will already be receiving training via their MTU, there exists the ability to coordinate timelines so that the general public also has the opportunity to sign up for modules such as the one described in this case. It overcomes the obstacle of having no trained facilitators in remote areas and maximizes the utility of the MTU. By providing increased training opportunities to both our medical and non-medical community members, we are creating a safer environment for our province as a whole.

Although hypothermia and drowning are the two key injuries that we focus on in this module, other areas that would benefit from offering public education opportunities include bone/joint injuries, wilderness survival or wound care.

## Conclusions

Increasing the public’s knowledge about how to assess and manage injuries that are common to our province increases the safety and well being of everyone. Further exploration into evaluating this training module and development of other ones should be investigated as opportunities to deliver training become more accessible.
